# Genetic control of astrocyte function in neural circuits

**DOI:** 10.3389/fncel.2015.00310

**Published:** 2015-08-17

**Authors:** Hannah M. Jahn, Anja Scheller, Frank Kirchhoff

**Affiliations:** Molecular Physiology, Center for Integrative Physiology and Molecular Medicine (CIPMM), University of SaarlandHomburg, Germany

**Keywords:** genetic targeting of astrocytes, tamoxifen, CreERT2/loxP, Bergmann glia, human GFAP promoter, GLAST glutamate aspartate transporter

## Abstract

During the last two decades numerous genetic approaches affecting cell function *in vivo* have been developed. Current state-of-the-art technology permits the selective switching of gene function in distinct cell populations within the complex organization of a given tissue parenchyma. The tamoxifen-inducible Cre/loxP gene recombination and the doxycycline-dependent modulation of gene expression are probably the most popular genetic paradigms. Here, we will review applications of these two strategies while focusing on the interactions of astrocytes and neurons in the central nervous system (CNS) and their impact for the whole organism. Abolishing glial sensing of neuronal activity by selective deletion of glial transmitter receptors demonstrated the impact of astrocytes for higher cognitive functions such as learning and memory, or the more basic body control of muscle coordination. Interestingly, also interfering with glial output, i.e., the release of gliotransmitters can drastically change animal’s physiology like sleeping behavior. Furthermore, such genetic approaches have also been used to restore astrocyte function. In these studies two alternatives were employed to achieve proper genetic targeting of astrocytes: transgenes using the promoter of the human glial fibrillary acidic protein (GFAP) or homologous recombination into the glutamate-aspartate transporter (GLAST) locus. We will highlight their specific properties that could be relevant for their use.

## Introduction

**Figure 1:**
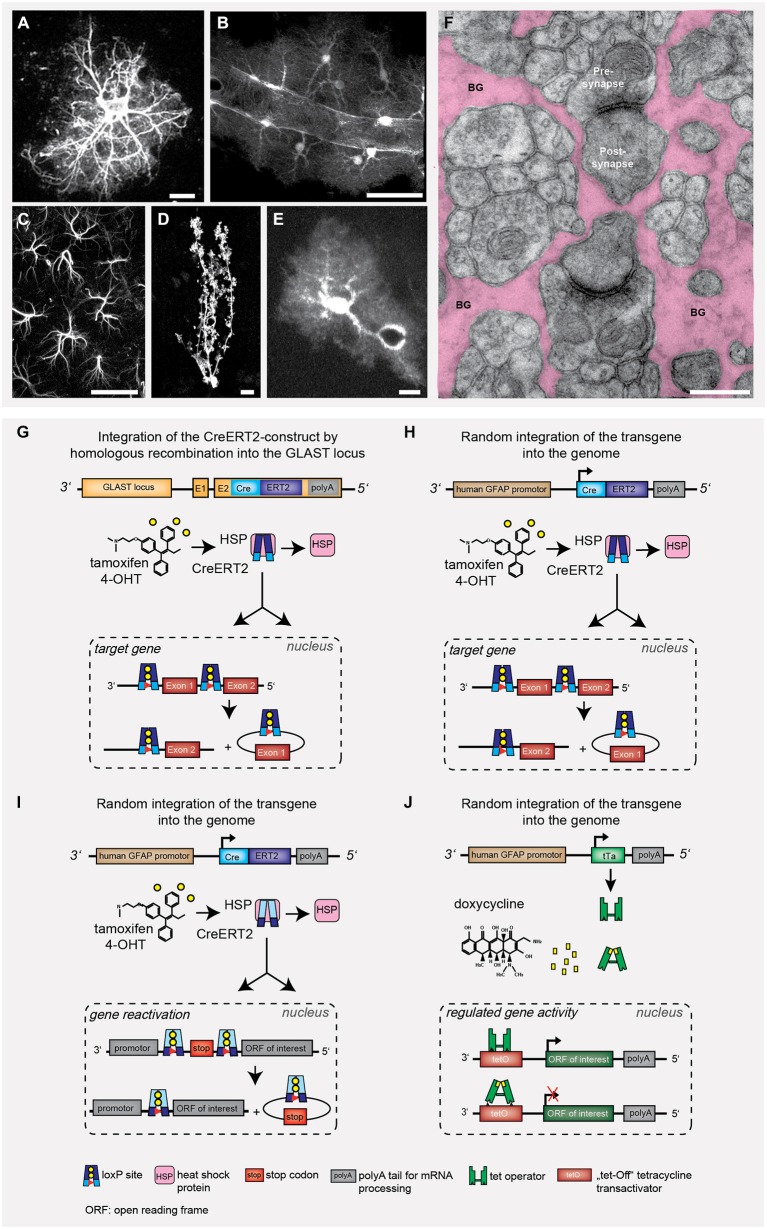
**Astrocyte heterogeneity and gene targeting strategies to influence astrocyte behavior.** Throughout development and in different brain regions, the heterogeneity of astrocytes becomes rather obvious when looking at the different morphologies. It is more the cell volume with cytosol and cell membrane that helps to visualize astrocyte function rather than the cytoskeletal structure **(A–F)**. Only few genetic strategies have been used to modify astrocyte function *in vivo* **(G–J)**. **(A)** glial fibrillary acidic protein (GFAP)-stained acutely isolated astrocyte. **(B)** Cortical astrocytes expressing tdTomato in close contact to a blood vessel with their end feet, **(C)** Hippocampal astrocytes (CA1) expressing GFAP. **(D)** Single Bergmann glia (BG) cell with CreERT2/loxP controlled reporter expression (EGFP). **(E)** Cortical astrocyte expressing EGFP and surrounding a blood vessel. Scale bars: **A,D,E** = 10 μm, **B** = 20 μm, **C** = 50 μm. **(F)** Electron micrograph depicting the intimate enwrapping of pre- and postsynaptic terminals by astroglial processes (BG: Bergmann glial processes, scale bar: 1 μm). **(G)** Knock-in of CreERT2 into the GLAST locus leads to tamoxifen-sensitive recombination in all astrocytes with endogenous GLAST promoter activity (Mori et al., 2006). The DNA recombinase variant CreERT2 is trapped in the cytosol by heat shock proteins (HSP), after tamoxifen application the protein is released and translocated into the nucleus. **(H,I)** Transgenic GFAP-CreERT2 mice generated by non-homologous recombination can also be used to target astrocytes (Hirrlinger et al., 2006). The Cre/loxP system can either be used to selective excise gene alleles of interest (**G,H**; knockout) or to express genes of interest (e.g., reporter proteins such as GFP or genetically encoded Ca^2+^ indicators), but also to restore gene function **(I)** (Lioy et al., 2011). **(J)** Alternatively, the binary tTA/tetO system composed of (1) promoter-controlled expression of a tetracyclin transactivator protein; and (2) tetracycline/doxycycline-responsive elements driving the expression of proteins-of-interest (Pascual et al., 2005). This system allows for a certain degree of reversible gene regulation.

Astrocytes represent an abundant, but also heterogeneous group of glial cells in all regions of the brain (Figures 1A–F). Their numerous interactions with capillaries and neurons are important signaling pathways for physiological brain function. Astrocytes actively control signal processing and transmission at the tripartite synapse (Perea et al., 2009). Originally, astrocytes were regarded as silent non-excitable cells, since they do not communicate via electrical signals. But now, it has become very clear that astroglial signaling is encoded in complex spatial and temporal patterns of Ca^2+^ changes within subcellular compartments as well as throughout cellular networks coupled by gap junctions. Intracellular Ca^2+^ rises indicate how they sense activity of their surroundings (Zorec et al., 2012; Araque et al., 2014; Verkhratsky and Parpura, 2014). Intracellular changes of another cation, Na^+^, have been recognized as an additional or alternative indicator of astrocyte activation (Kirischuk et al., 2012; Verkhratsky et al., 2013; Rose and Chatton, 2015). Even over longer distances astrocytes can convey various signals, e.g., inositol-1,4,5-trisphosphate (IP3) or cyclic nucleotides that pass through gap junctions and functionally couple the astroglial syncytium (Giaume and Liu, 2012; Theis and Giaume, 2012). Within these networks information spreads with tens of μm/s speed, still several orders of magnitude slower than the propagation of neuronal action potentials (Haydon and Nedergaard, 2015). The interactions of astrocytes with neurons are largely based on astroglial receptors that sense neuronal communication or on the release of gliotransmitters acting back on synaptic transmission. The general importance of astrocytes for brain function is uncovered by the devastating point mutations in single genes encoding transcription factors since these factors can control extended sets of gene programs. Genetically modified mice addressing astrocyte function have been instrumental in uncovering their role in the living animal.

In general, transgenic mouse models that interfere with cell function can be categorized in three classes: (1) Modulation of information input by direct knockout of receptors or signaling molecules, thereby preventing the information to enter the cell; it becomes *insensitive*; (2) Cell *perturbation* of intracellular gene programs or metabolic pathways, e.g., by knockout of transcription factors or enzymes involved in signal processing and resulting changes of the cellular infrastructure; and (3) Inhibition of signal release to the adjacent cellular neighborhood; the astrocyte becomes *inactive* or *silent*.

Conditional gene deletions started with the use of cell-specific promoters driving the Cre DNA recombinase (Cre/loxP system) to abolish gene function in selected cell types. However, several of such regulatory elements were active in precursor cells during embryonic development, thereby affecting complete lineage trees. To achieve temporal control of cell-specific DNA recombination, tamoxifen-sensitive, i.e., inducible variants of the Cre DNA recombinase were developed (CreER, CreER^T^, CreER^T2^). Here, the DNA recombinase Cre is fused to a mutated ligand-binding domain of the human estrogen receptor (ER), which does not bind endogenous estradiol, but is highly sensitive to nanomolar concentrations of 4-hydroxytamoxifen, a metabolite of tamoxifen that can be applied intraperitoneally, but also by gavage (Feil et al., 1997; Metzger and Chambon, 2001; Weber et al., 2001; Hirrlinger et al., 2006; Mori and Zhang, 2006).

Here, we will review current genetic strategies to reveal the impact of astrocyte function in the brain by focusing on frequently used paradigmatic examples (Figures 1G–J): tamoxifen-sensitive cell-specific gene deletion and restoration as well as doxycycline-induced expression of functionally impaired, dominant-negative signaling molecules. The tamoxifen-sensitive CreERT2/loxP system provides temporal control of gene deletion (Hirrlinger et al., 2006; Mori et al., 2006), in contrast to conventional mouse models with Cre/loxP (Malatesta et al., 2003). The doxycycline/tTA model can be used for transient effector protein expression (Bujard, 1999; Mansuy and Bujard, 2000; Pascual et al., 2005).

In the first part we will shortly describe the mouse models and their associated astrocyte function. In the second part, we will compare the targeting of astrocytes using regulatory elements of the human glial fibrillary acidic protein (GFAP) promoter with expression from the GLAST gene locus directly, and deduce respective technical considerations and scientific limitations.

## The Role of Astrocytes in Hippocampal Learning

**Figure 2:**
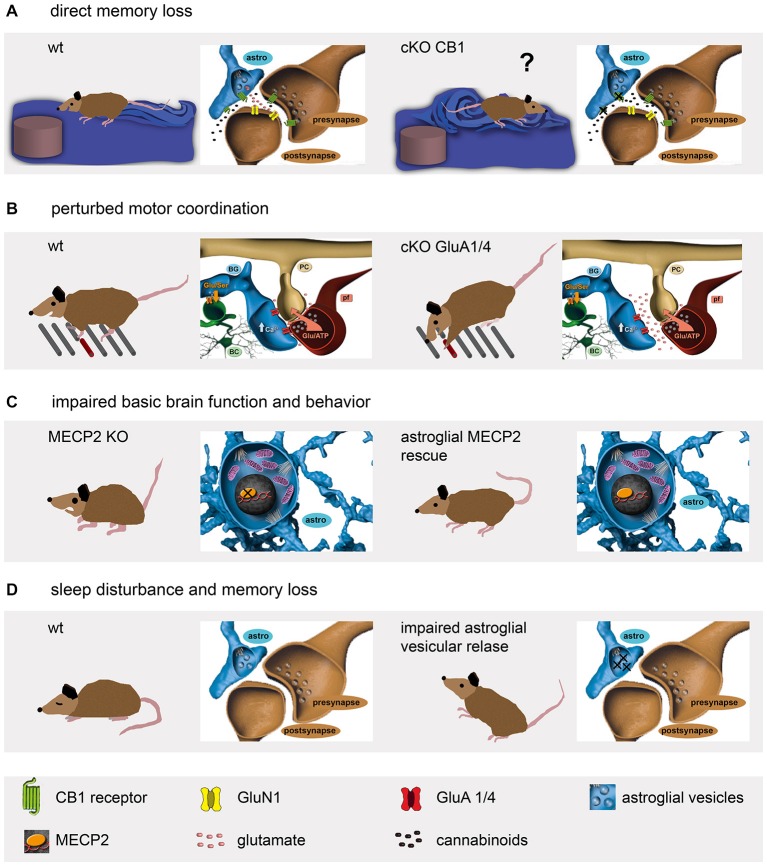
**Genetic mouse models reveal a diversity of astrocyte functions affecting mouse behavior. (A)** Astroglial cannabinoid receptors are involved in spatial memory formation (Han et al., 2012). **(B)** The ionotropic glutamate receptors on BG contribute to fine motor coordination (Saab et al., 2012). **(C)** Although the lack of the widely expressed transcriptional repressor MECP2 results in synapse loss, severe mental retardation and premature death, the astrocyte-specific re-expression restores several vital functions like motor activity (Lioy et al., 2011). **(D)** Impairment of astroglial ATP release perturbs sleep behavior and induces memory loss (Pascual et al., 2005; Halassa et al., 2009).

The functional role in working memory and its molecular signaling of the hippocampal CA3-CA1 synapse have been well characterized (Neves et al., 2008; Bannerman et al., 2014). A diversity of endogenous compounds is capable of modulating its transmission and thereby affecting our memory. Interestingly, one of the most abundant G-protein coupled receptors of the brain, the cannabinoid type-1 receptor (CB_1_R), has been detected on all hippocampal cell types (Marsicano and Lutz, 1999). Based on work performed on brain slices, endocannabinoids or the synthetic drug Δ9-tetrahydrocannabinol (THC), better known as marijuana, are thought to inhibit presynaptic transmitter release via CB_1_R activation, thereby depressing excitatory neurotransmission, and finally, impairing spatial working memory (Misner and Sullivan, 1999; Carlson et al., 2002; Takahashi and Castillo, 2006; Bajo et al., 2009; Schoeler and Bhattacharyya, 2013). Also *in vivo* THC was found to cause long-term depression (LTD; Hampson and Deadwyler, 2000; Mato et al., 2004; Madroñal et al., 2012). However, when the impact of CB_1_R was tested more selectively using genetically modified mice with cell-specific receptor deficiency, unexpectedly, it was the ablation of the astroglial (Figure 1H; GFAP-CreERT2 × floxed CB_1_R), but not the neuronal CB_1_R that completely abolished THC-dependent depression (Figure 2A; Han et al., 2012). For their analysis, the authors used mice with at least 4 weeks of time to allow efficient receptor protein degradation. Although 30% of CB_1_R protein could still be detected using immune-EM, the receptor function in modulating synaptic efficacy, i.e., LTD, was completely gone. In parallel, the mutant mice remained unaffected after THC injection when behaviorally tested in a variant of the Morris water maze. Thereby, this study demonstrates the pivotal role of astrocytes in modulating synaptic transmission and respective circuit-associated learning behavior.

## Cerebellar Bergmann Glia Fine Tune Neural Circuits of Motor Coordination

The neural circuits of the cerebellum control the timing of motor performance by integrating sensory and motor input of climbing and mossy fibers (De Zeeuw et al., 2011; Heck et al., 2013). While climbing fibers (CF) multiply innervate the principal neurons, i.e., the Purkinje cells (PC); the information from mossy fibers is relayed via granule cells and their parallel fibers (PF). Both synapses, CF-PC and PF-PC, are characterized by their almost complete coverage by membrane appendages emanating from the main radially oriented processes of the Bergmann glia (BG; Figures 1D,F; Grosche et al., 2002; Lippman et al., 2008; Lippman Bell et al., 2010). The perisynaptic glial membranes are equipped with a tremendously high density of glutamate transporter to strictly control the extracellular levels of the excitatory transmitter and preventing synaptic spillover (Tong and Jahr, 1994; Marcaggi et al., 2003; Takayasu et al., 2006, 2009). Surprisingly, BG processes were found to express another glutamate sensing mechanism, ionotropic glutamate receptors very similar to neurons (Müller et al., 1992). The heteromeric ion channel complexes are formed by an assembly of the AMPA-type receptor subunits GluA1 and GluA4 (Geiger et al., 1995). However, in contrast to several neuronal AMPA receptors, the BG receptor channels are Ca^2+^ permeable since they lack expression of the GluA2 subunit. While the importance of efficient glutamate uptake at excitatory synapses appears quite obvious, the expression of fast desensitizing AMPA receptors on BG remained enigmatic till very recently. To completely abolish AMPA receptor function in BG, another astrocyte-specific mouse, the GLAST-CreERT2 knockin (gene: *slc1a3*; Mori et al., 2006; Figures 1G, 2B) was used to delete GluA1 and GluA4 simultaneously (Saab et al., 2012). In young mice (2 to 4 weeks old) GluA1/4 deletion resulted in retraction of glial appendages from PC synapses, an increased amplitude and duration of evoked postsynaptic PC currents, and a delayed formation of glutamatergic synapses. In adult (older than 2 months) mice, GluA1/4 inactivation also caused retraction of glial processes. In addition, the study provided a detailed time course for tamoxifen-evoked gene excision as well as mRNA and protein degradation. While the recombination event was almost completed after 2 days, the mRNA persisted for a week and the protein for even 3 weeks. The mutant mice showed normal behavior when observed in their cages. However, when the mice were challenged by a complex motor task running along a horizontal ladder with suddenly appearing obstacles, their fine motor coordination was significantly impaired (Figure 2B). Thus, AMPA receptors of BG are essential to optimize synaptic integration and cerebellar output function throughout life. AMPA receptor signaling of BG contributes to the structural and functional integrity of the cerebellar network and plays an important role in the “fine-tuning” of neuronal processing, which is crucial for a fast and precise control of complex motor behaviors (Saab et al., 2012).

## Astrocyte-Specific Intracellular Gene Regulation in Signal Conversion

The above examples demonstrate how important astrocytes are in extracellular sensing of neuronal activity. Important intracellular functions such as detoxification or gene regulation can already be significantly affected by single point mutations in genes encoding enzymes or transcription factors, as observed in a variety of neurological disorders. A particularly well studied example is the methyl-CpG binding protein 2 (MECP2) that acts as a transcriptional repressor and affects the activity of broad range of downstream genes (Chahrour and Zoghbi, 2007). Mutations of the X-linked MECP2 gene cause Rett syndrome, a neurodevelopmental disorder. At the end of infancy patients suffer from progressing synaptic malfunctions associated with severe mental retardation (Chahrour and Zoghbi, 2007). Since loss-of-function mutations in mice phenocopy the human disease, genetic rescue experiments were performed to re-introduce the fully functional wild type gene. And indeed, global gene rescue restored brain development and function (Guy et al., 2007). Interestingly, neuron-specific restoration prevented several, but not all Rett-associated symptoms (Alvarez-Saavedra et al., 2007). Since MECP2 can be detected in astrocytes as well, it was selectively re-introduced in astrocytes using GFAP-CreERT2 mice crossbred to genetically modified mice with the MECP2 open reading frame encoded by the endogenous gene, but separated from the promoter region by a Cre-excisable STOP fragment (Figures 1I, 2C; Lioy et al., 2011). Tamoxifen treatment of mutant mice prolonged the lifespan to 7.5 months when compared to oil-treated animals that already died at 3 months of age. Phenotype differences became evident 6 weeks after tamoxifen-induced MECP2 re-expression. Even highly symptomatic MeCP2^stop/y^ mice could be rescued. While MECP2-deficient mice were hyperactive and quite anxious, behavioral testing in the open field and water maze revealed an improvement to 50% of control levels (Figure 2C). Similar to Rett patients, 12-week-old MeCP2^stop/y^ mice displayed strong breathing problems, but 2 months after tamoxifen-induced rescue the respiration pattern recovered to a normal range. Interestingly, the astrocyte-specific deletion of MeCP2, induced at an age of 3 weeks, caused several pathological symptoms such as smaller body size, clasped hindlimb posture and irregular breathing 10 weeks later as observed in the global knockout mice (Han et al., 2012). Lifespan, locomotion and anxiety behavior, however, remained normal. Thereby, these data suggest distinct mechanisms of neuron–astrocyte interactions in different neural circuits of forebrain (anxiety, motor behavior) and hindbrain (control of respiration).

## Astrocytes Modulate Synaptic Transmission

One genetic mouse model in glia research has received particular attention and is currently heavily debated, the doxycycline-switchable dnSNARE mouse (Pascual et al., 2005; Sloan and Barres, 2014; Haydon and Nedergaard, 2015; Figures 1J, 2D). In this paragraph we will provide only a description of the positive findings that have been made. In the last part of this section we will shortly address the current discussion.

A very clear and evident way how astrocytes affect neural circuits became obvious when the regulated secretion of gliotransmitters such as glutamate, ATP or D-serine was investigated (Pascual et al., 2005). For this purpose a different switchable genetic mouse model was developed: the tetracycline-dependent expression of dominant-negative (dn) soluble N-ethylmaleimide-sensitive factor attached receptor (SNARE)-proteins that disrupt the vesicular release of gliotransmitters. Transgenic mice with human GFAP promoter-driven expression of the *tetoff* tetracycline transactivator (tTA; Mansuy and Bujard, 2000) were crossbred to mice with a tTA responsive element (tet operator, tetO) regulating expression of dnSNARE and reporter proteins (Figure 1J). In the presence of dietary doxycycline, a tetracycline derivative with improved permeability for the blood-brain barrier, the expression of dnSNAREs and reporter proteins is suppressed. Two weeks after stopping doxycycline, astrocytes efficiently express dnSNAREs and block their vesicular release of gliotransmitters (Figure 2D), in particular of ATP that is further degraded to adenosine. And indeed, electrophysiological analysis of hippocampal slices reveal enhanced field potentials after Schaffer collateral stimulation in dnSNARE mice (Pascual et al., 2005). Extracellular adenosine, generated from released ATP, acts on presynaptic A1 receptors and suppresses excitatory synaptic transmission. Blocking the constitutive ATP/adenosine release from astrocytes enhances the excitatory drive that can be blocked by exogenous application of ATP (Pascual et al., 2005). By releasing activity dependent neurotransmitters, astrocytes regulate the strength of basal synaptic transmission at the circuit level. At system and behavioral level, impaired astroglial ATP release reduces the slow wave activity in the electroencephalogram (EEG), perturbs sleep homeostasis and sleep loss-associated memory deficits (Figure 2D; Halassa et al., 2009).

In a recent study severe concerns about the use of the transgenic dnSNARE mice have been raised (Fujita et al., 2014). The authors criticize an insufficient early characterization of the mice and present own data that suggest a predominant neuron-mediated impairment of ATP signaling rather than an astroglial one. Without joining this discussion, it is evident that far-reaching conclusions from genetically engineered animal models should be based on independent experimental systems. But this should be immanent to all types of science. Continuous generation of novel mouse models and their free sharing within the scientific community will warrant the progress in our understanding of brain function.

## Technical Considerations When Using Genetically Modified Mice to Target Astrocyte Functions

**Figure 3:**
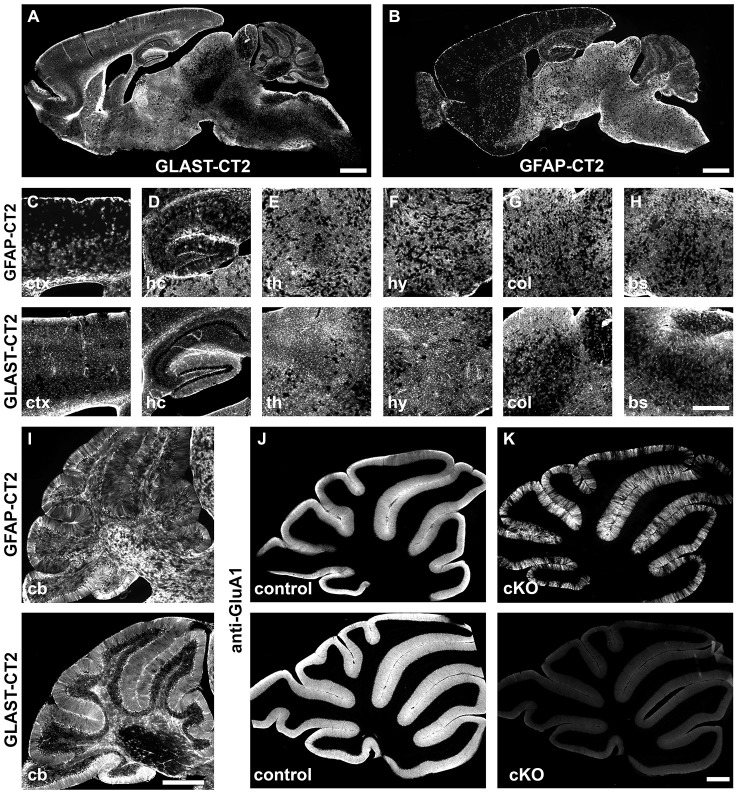
**Comparison of tamoxifen-induced DNA recombination in GLAST-CreERT2 and GFAP-CreERT2 mice.** Comparison of DNA recombination in transgenic GFAP-CreERT2 and GLAST-CreERT2 knockin mice. **(A,B)** Sagittal overview of tdTomato reporter expression (Madisen et al., 2010) in the brain of GLAST-CreERT2 and GFAP-CreERT2 mice. **(C–H)** Magnified views of selected brain regions (ctx, cortex; hc, hippocampus; th, thalamus; hy, hypothalamus; col, superior colliculus; bs, brainstem). The upper panel shows reporter activation in GFAP-CreERT2 mice, the lower panel in GLAST-CreERT2. **(I–K)** In the cerebellum, tdTomato reporter activation of BG and other astrocytes is comparable in GFAP-CreERT2 and GLAST-CreERT2 mice **(I)** however, gene deletion (here GluA1) is more efficient **(K)** in BG of GLAST-CreERT2 mice (lower panel) than in GFAP-CreERT2 mice (upper panel) when compared to control mice **(J)**.

Astrocytes are not only widely distributed throughout all regions of the brain, age and brain-region specific expression of genes and their distinct functions have been identified (Malatesta et al., 2003; Regan et al., 2007; Halassa et al., 2009; Robel et al., 2009, 2011; Gourine et al., 2010; Lioy et al., 2011; Han et al., 2012; Saab et al., 2012). Therefore, the selection of regulatory elements of THE astrocyte-specific gene is almost impossible. Historically, the promoter of the human GFAP gene has become a widely distributed and valuable tool. Its small size of 2.2 kb offered excellent cloning properties and facilitated its use for transgenic expression of numerous proteins of current interest such as EGFP or CreERT2 (Nolte et al., 2001; Hirrlinger et al., 2006). In parallel, as an alternative genetic tool, GLAST-CreERT2 mice were generated by targeting the tamoxifen-sensitive Cre DNA recombinase CreERT2 to exon 2 of the GLAST locus using homologous recombination (Mori et al., 2006). Direct comparison of both mouse lines revealed variable differences in brain region-dependent recombination although they largely overlap (Figure 3). While recombination in GLAST-CreERT2 mice dominates forebrain regions, the GFAP-CreERT2 mouse displays higher recombination efficiencies in the hindbrain. Particular differences become evident when not only reporter proteins are activated, but when gene knockout experiments require the recombination of homozygous alleles as observed for the deletion of the ionotropic glutamate receptor subunit GluA1 in cerebellar BG (Figures 3J,K). In GLAST-CreERT2 mice the immunohistochemical signal indicating GluA1 was completely abolished to background levels 4 weeks after induction of recombination, while in GFAP-CreERT2 mice GluA1 expression still remained in numerous BG cells.

It is important to note that only heterozygous GLAST-CreERT2 mice can be used. Homozygous GLAST-CreERT2 mice will be knockouts of the GLAST-dependent glutamate uptake. Interestingly, we found that, although heterozygous mice express only 50% of GLAST mRNA and protein in comparison to wild type mice, the functional glutamate uptake current appears to be unaffected (Saab et al., 2012).

Although the GFAP-CreERT2 and GLAST-CreERT2 mice have been successfully used in a series of studies including behavioral experiments, both suffer from recombination in radial glia of the neurogenic niches (Hirrlinger et al., 2006; Mori et al., 2006; DeCarolis et al., 2013). The DNA recombination induced in the respective neuronal progeny might contribute to a phenotype in conditional knockout experiments and careful control experiments should be employed. Alternative lines such as Connexin43-CreERT mice (Eckardt et al., 2004) without neuronal recombination in stem cells have not yet been used for behavioral experiments. Similarly, behavioral experiments with astrocyte-specific AldhL1- or FGFR3-CreERT2 mice might also be difficult since recombination can also be induced in neural stem cells or interneurons (Young et al., 2010; Yang et al., 2011; Foo and Dougherty, 2013).

Tamoxifen-induced gene recombination is a very valuable tool to temporally control gene excision. But, one has to keep in mind that gene excision does not mean simultaneous disappearance of the respective protein. The half-lifes of mRNA and protein have to be considered. In case of the glutamate receptors GluA1 and GluA4, their efficient functional removal required about 2 weeks (Saab et al., 2012). In addition, the age of the mice (i.e., the level of gene activity) influenced the protein turnover as well.

In the future, genetic targeting of astrocytes has to become more sophisticated. It is now clear that astrocytes of different brain regions can fulfill different functions. Therefore, we have to identify novel regulatory elements that can be used to address the astroglial heterogeneity, either by targeting astrocytes of distinct brain regions locally or functionally, e.g., after injury or during a learning paradigm. The numerous transporter genes could be a rich source. The thyroid hormone transporter OATP1C1 is such an example. This transporter is expressed by cortical or hippocampal astrocytes, but not in the brainstem (Schnell et al., 2013). In addition, the coincident use of two different gene loci and employing the split-Cre system could provide another strategy to target subclasses of astrocytes (Hirrlinger et al., 2009a,b).

Current efforts in obtaining cell type, age and brain region-dependent gene expression profiles will facilitate the quest for suitable regulatory elements to target astrocytes. Future mouse models will then help to further highlight selective astrocyte function of distinct central nervous system (CNS) regions or learning paradigms.

## Conflict of Interest Statement

The authors declare that the research was conducted in the absence of any commercial or financial relationships that could be construed as a potential conflict of interest.
